# An improved *Escherichia coli* strain to host gene regulatory networks involving both the AraC and LacI inducible transcription factors

**DOI:** 10.1186/1754-1611-8-2

**Published:** 2014-01-02

**Authors:** Manjunatha Kogenaru, Sander J Tans

**Affiliations:** 1 FOM Institute for Atomic and Molecular Physics, Science Park 104, 1098 XG Amsterdam, The Netherlands; 2 Present address: EMBL/CRG Systems Biology Research Unit, Centre for Genomic Regulation (CRG), Dr. Aiguader 88, 08003 Barcelona, Spain; 3 Present address: Universitat Pompeu Fabra (UPF), 08003 Barcelona, Spain

**Keywords:** Escherichia coli, Strain, Gene network, Transcription factor, Repressor, Regulatory cascade

## Abstract

Many of the gene regulatory networks used within the field of synthetic biology have extensively employed the AraC and LacI inducible transcription factors. However, there is no *Escherichia coli* strain that provides a proper background to use both transcription factors simultaneously. We have engineered an improved *E. coli* strain by knocking out the endogenous *lacI* from a strain optimal for AraC containing networks, and thoroughly characterized the strain both at molecular and functional levels. We further show that it enables the gradual and independent induction of both AraC and LacI in a simultaneous manner. This construct will be of direct use for various synthetic biology applications.

## Introduction

Synthetic biology often deals with engineering of biological circuitry to obtain desired phenotypes. The successful design and construction of the first synthetic gene circuits like the genetic toggle switch [1] and the repressilator [2] have demonstrated that engineering-based methodology could indeed be used to build sophisticated, computing-like behavior into biological systems. Well-understood transcription factors are the key element for such circuits. Although many different transcription factors are found in nature, majority of them have not been well-characterized, and even their performance may be unpredictable [3]. Further, it is becoming evident that the host strain has a profound influence on the synthetic gene circuits phenotypic response [4]. Hence synthetic biology field has extensively used just a few well-characterized parts, in particular the arabinose operon regulator (AraC), lactose operon regulator (LacI), tetracycline operon regulator (TetR*)* and λ phage regulator (CI).

A plethora of *E. coli* strains is available to harbor synthetic regulatory networks built from TetR and CI parts, owing to their exogenous nature. However, the choice is limited for the networks that use the LacI and AraC parts, as they are endogenous to *E. coli*. Currently, there are ~80 strains available with a *lacI* mutation at the coli genetic stock center, which may in principle be used to harbor constructs with the LacI, TetR and CI. For applications that aim to combine the AraC and LacI*,* one may consider strains KL390 (CGSC#6207), KL384 (CGSC#6205) and KL385 (CGSC#6206), since they have mutations in both the *araC* and *lacI*. However, these strains lead to an all-or-none response by the *araBAD* promoter (P_BAD_), in which cells either display full expression or only basal expression, while gradual induction cannot be achieved, especially at the low concentration of the L-Arabinose inducer [5]. This all-or-none phenomena occurs because, expression of the inducer transporter is controlled by the inducer itself [5]. Additionally, these strains contain arabinose metabolizing genes, which results in the decrease of the effective concentration of L-Arabinose as the cells utilize it.

Here we constructed a host suitable for gene regulatory networks involving both the AraC and LacI transcription factors. We used the strain BW27783 (CGSC#12119) as a basis, since it carries a deletion for the arabinose metabolizing genes, and also abolished the all-or-none response, with a copy of the low-affinity high-capacity transporter, *araE,* under the control of an arabinose-independent promoter [6]. To allow for networks containing LacI as well, we knocked out the endogenous *lacI* copy from the BW27783 strain, using lambda Red recombinase mediated site-specific genome engineering technology [7]. We characterized the resulting new strain for the possible effects on the growth rate, and further show that it allows for the gradual and independent induction of AraC and LacI.

## Results and discussion

The parent strain BW27783 carries a ∆*lacZ*4787(::*rrnB-3*) mutation, consisting of three tandem copies of *rrnB* transcriptional terminators inserted within the promoter of the lactose (*lac*) operon [6]. This suppresses the expression of *lac* operon, which was confirmed by the LacZ assay (see Additional file 1: Figure S1). Hence deletion of the *lacI* in this strain will not lead to the over expression of the *lac* operon.

**Figure 1:**
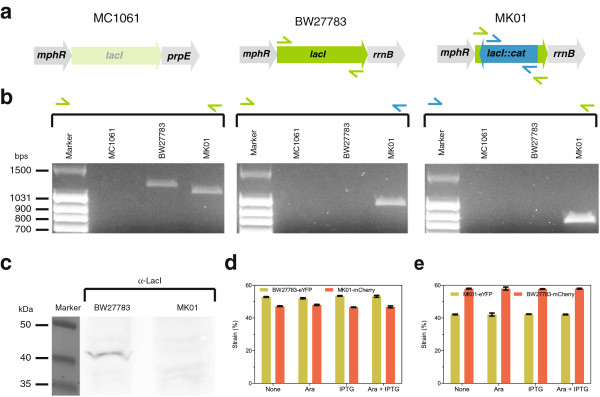
**Characterization of the engineered strain. (a)** Schematic representation of the genotype at the engineered locus is shown for MC1061 negative control, BW27783 parent strain, and MK01 derived strain. **(b)** This panel shows PCR verification of engineered strain using different primer pairs for negative, parent and derived strains as templates. Left panel shows PCR amplicons resulting from the primers specific to the flanking regions, whereas the middle and right panel show the primers specific to the flanking and inserted selection cassette. Marker bands are shown in bps. **(c)** Western blot of parent and engineered strains using anti-LacI antibody is displayed. Maker bands are shown in kDa. **(d)** This panel shows results of a competition assay, showing the percentage of the parent and engineered strains after mixing them at equal ratios and growth during 15 hours. The data were taken using flow cytometry and labeling of the two strains with different fluorescent markers. **(e)** Same as **(d)**, but with swapped fluorescent reporters: BW27783-mCherry and MK01-eYFP.

We replaced the *lacI* with chloramphenicol acetyltransferase (*cat*) selection cassette in the parent strain BW27783, which resulted in the engineered strain MK01. We further confirmed the resulting *lacI*::*cat* replacement mutation in the MK01 strain by performing three independent PCR reactions (Figure 1). The first reaction was carried out with the primer pair specific to the flanking regions of the inserted selection cassette that was not affected by the replacement mutation. Therefore, this primer set produced PCR amplicons with both the parental BW27783 as well as the engineered MK01 strains. The expected band size for BW27783 was 1209-base pairs (bps), whereas for MK01 it was 1123-bps, and the difference of 86-bps was quite evident on the gel (Figure 1b, left). We further used the *E. coli* strain MC1061, as a negative control, which carries a complete deletion for *lac* operon, due to the mutation ∆*(codB-lacI)3 *[8], thereby no PCR amplicons were produced (Figure 1b). Additionally, two more PCR reactions were performed using the above flanking primers with an additional primers specific to the inserted selection cassette (Figure 1a, right). These reactions produced amplicons only with the MK01 template as expected (Figure 1b, middle and right). Depending on the primer sets used, the expected band size was either1020-bps (Figure 1b, middle) or 850-bps (Figure 1b, right). We further confirmed the *lacI* deletion by sequencing the genomic PCR amplicons from the MK01 template (data not shown). Sequencing results together with the PCR verification confirmed that *lacI* was successfully replaced by a selection cassette, with proven correct new junctions. We also chose to retain the *cat* resistance gene from the MK01 strain, as it serves as a good phenotypic marker for the *lacI* deletion mutation. When required, the selection cassette can be easily eliminated with the help of Cre recombinase, derived from a bacteriophage P1 that recombines the LoxP sites on either side of a selection cassette, thereby resulting in the excision of the selection cassette.

In order to further confirm the *lacI* deletion at the level of protein, we carried out Western blot analysis on both the parent and engineered strains (Figure 1c). This confirms that the parent strain indeed expresses *lacI* repressor, as the protein was detected around 40 kilo Dalton (kDa). In particular, the engineered strain MK01 didn’t show any detectable protein around that size, confirming the complete knockout of the *lacI* repressor (Figure 1c).

To assess the possible effects on the growth, we measured the relative accumulation of the parental and engineered cells in a stationary-phase culture. For this, we labeled these cells with two different fluorescent proteins (eYFP and mCherry), using constitutively expressing constructs (see Additional file 1: Figure S2a and b), and performed competition experiment in M9 minimal medium. We initiated the competition by mixing equal densities of the cultures from both the strains and measured their relative abundance after 15 hours of the growth. This assay revealed that the engineered MK01 strain accumulated 5.6% less than the parent BW27783 strain (Figure 1d). After swapping the fluorescent protein marker, MK01 again accumulated less than the parent strain (15.8%, Figure 1e). These observations could be attributed to the constitutive expression of the *cat* selection cassette used to replace the *lacI*. Additionally, maintenance of the whole reporter plasmids used to label the strains may also influence the growth rates [9]. Furthermore, addition of the inducers L-Arabinose and Isopropyl β-D-1-thiogalactopyranoside (IPTG) did not show any differences in the relative accumulation, consistent with the idea that these inducers are non-metabolizable (Figure 1d and e).

**Figure 2:**
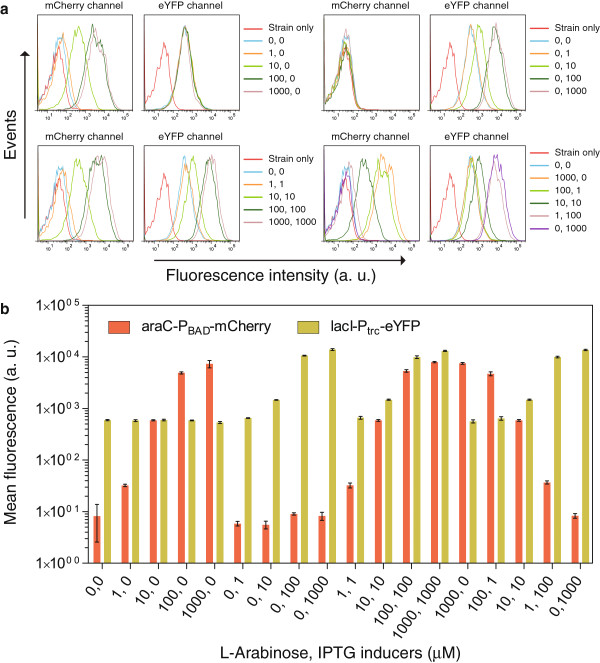
**Gradual and independent induction of AraC and LacI. (a)** Histograms showing the single-cell fluroscence intensities (arbitrary units) as measured by flow cytometry, using the engineered MK01 strain. The strain harbored two plasmids expressing mCherry and eYFP fluorescent reporters under the control of the AraC and LacI respectively. The concentrations of the inducers, L-Arabinose and IPTG used in each conditions are indicated. One representative replicate from the multiple replicates is shown for each conditions. **(b)** The mean fluorescence intensities of the data displayed in panel **a**. The error bars indicate the std. dev. of 3–5 replicates. Other labels are as for Figure 2a.

Finally, to demonstrate the usefulness of the engineered MK01 strain in hosting the constructs involving both the AraC and LacI transcription factors simultaneously, we transformed this strain with the constructs on two different plasmids (see Additional file 1: Figure S3a and b), in which the expression of *mCherry* and *eYFP* target reporter genes are regulated by these transcription factors. The data showed a gradual increase in the expression of the reporters with increasing concentrations of L-Arabinose and IPTG inducers (Figure 2). For this wide range of inducers concentrations, the population of cells responded homogeneously (Figure 2a). Further, the AraC-controlled expression obtained at different concentrations of L-Arabinose remained same within the measurement error in both presence and absence of the LacI inducer IPTG (Figure 2b). Similarly, the LacI-controlled expression obtained at different concentrations of IPTG was not influenced by the AraC inducer L-Arabinose. Thus, these transcription factors could be induced gradually and independently. Further our induction assay shows that AraC modulates the target gene expression up to 898-fold, whereas, LacI modulates its target gene up to 23-fold (Figure 2b). Overall this demonstrates that our engineered MK01 *E. coli* strain is well-suited to study the gene regulatory networks involving both the AraC and LacI transcription factors simultaneously in a more quantitative manner.

## Materials and method

The strain BW27783 (genotype: F-, ∆(*araD-araB*)567, ∆*lacZ*4787(::*rrnB-3*), *λ-*, ∆(*araH*-*araF*)570(::FRT), ∆*araEp*-532::FRT, φP_cp8_-*araE535*, *rph-1*, ∆(*rhaD*-*rhaB*)568, *hsdR*514) [6] was used as a parental strain to derive the MK01 strain by following one-step inactivation of chromosomal genes procedure [7]. More detailed procedure on strain engineering can be found in the Additional file 1.

The expression of LacI protein was assayed by Western blotting using a mouse anti-LacI monoclonal antibody (Abcam), and a sheep anti-mouse secondary antibody (Jackson ImmunoResearch) conjugated to horseradish peroxidase.

Strains transformed with various DNA constructs (Additional file 1) were grown overnight in M9 minimal medium with appropriate antibiotics at 37°C. These cultures were further diluted to an optical density of 0.001 at 550 nm in the measuring plate. After 6 to 15 hours of further growth, cells were measured for fluorescence, using a BD LSRFortessa™ cell analyzer flow cytometer. The eYFP fluorescence was measured using a 488 nm excitation laser and a 515–545 nm emission filter, while mCherry was measured using a 561 nm excitation laser and 600–620 nm emission filter. A minimum of 10,000 cells was measured from each sample. From the single-cell fluorescence intensities, the mean fluorescence intensity per cell, representing the population average was calculated.

## Competing interests

The authors declare that they have no competing interests.

## Authors’ contributions

MK designed the study, performed the experiments and wrote the manuscript. SJT provided overall guidance and participated in writing the manuscript. Both authors read and approved the final manuscript.

## Supplementary Material

Additional file 1 **This document contains a detailed description of the methods along with the additional data and the DNA sequences of the constructs. Figure S1**. LacZ assay of engineered and parent strains along with the positive and negative controls. Luria-Bertani (LB) agar plates were coated with 40 μl of 20 mg/ml of 5-bromo-4-chloro-3-indolyl-β-D-galactopyranoside (X-Gal) and 40 μl of 100 mM IPTG. These plates were inoculated with cultures grown in LB medium at 37°C overnight. **(a)** Wild-type *E. coli* strain MG1655 that expresses functional β-galactosidase from the *lac* operon, hence results in blue colonies. **(b)** TOP10 strain contains ∆*lacZM15* mutation that encodes only *lacZ-ω* component, hence cannot produce blue colonies. **(c)** Parent strain BW27783 confirms that *lac* operon was not expressed, hence only white colonies are seen. **(d)** Engineered MK01 has also inherited the non-expressing *lac* operon mutation. **Figure S2**. The DNA sequences of the constructs used in the competition assay to estimate the relative accumulation. **(a)** This construct constitutively expresses the *eYFP* fluroscent reporter under the promoter *lacI*^*q*^. **(b)** This construct is same as S2a, but expresses *mCherry*, instead of *eYFP*. **Figure S3**. The DNA sequences of the constructs are shown used in the gradual and independent induction of AraC and LacI. **(a)** This construct constitutively expresses the *araC* from a bi-direction promoter *P*_*BAD*_, which inturn represses the transcription of the downstream *mCherry* fluroscent reporter. This repression can be relieved by the small-molecule inducer L-Arabinose. **(b)** This construct constitutively expresses the *lacI*, under the promoter *P*_*lacI*_, which in turn represses the expression of *eYFP* fluroscent reporter under the promoter *trc*, that can be relieved by the another small-molecule inducer IPTG. Click here for file
