# Supplementary material for: An improved Escherichia coli strain to host gene regulatory networks involving both the AraC and LacI inducible transcription factors
Source: J Biol Eng. 2014 Jan 2;8:2. doi: 10.1186/1754-1611-8-2 (PMC3881499; doi:10.1186/1754-1611-8-2)
Supplement: Additional file 1 — This document contains a detailed description of the methods along with the additional data and the DNA sequences of the constructs. Figure S1. LacZ assay of engineered and parent strains along with the positive and negative controls. Luria-Bertani (LB) agar plates were coated with 40 μl of 20 mg/ml of 5-bromo-4-chloro-3-indolyl-β-D-galactopyranoside (X-Gal) and 40 μl of 100 mM IPTG. These plates were inoculated with cultures grown in LB medium at 37°C overnight. (a) Wild-type E. coli strain MG1655 that expresses functional β-galactosidase from the lac operon, hence results in blue colonies. (b) TOP10 strain contains ∆lacZM15 mutation that encodes only lacZ-ω component, hence cannot produce blue colonies. (c) Parent strain BW27783 confirms that lac operon was not expressed, hence only white colonies are seen. (d) Engineered MK01 has also inherited the non-expressing lac operon mutation. Figure S2. The DNA sequences of the constructs used in the competition assay to estimate the relative accumulation. (a) This construct constitutively expresses the eYFP fluroscent reporter under the promoter lacIq. (b) This construct is same as S2a, but expresses mCherry, instead of eYFP. Figure S3. The DNA sequences of the constructs are shown used in the gradual and independent induction of AraC and LacI. (a) This construct constitutively expresses the araC from a bi-direction promoter PBAD, which inturn represses the transcription of the downstream mCherry fluroscent reporter. This repression can be relieved by the small-molecule inducer L-Arabinose. (b) This construct constitutively expresses the lacI, under the promoter PlacI, which in turn represses the expression of eYFP fluroscent reporter under the promoter trc, that can be relieved by the another small-molecule inducer IPTG. [file 1754-1611-8-2-S1.pdf]

Additional file 1

An improved *Escherichia coli* strain to host gene regulatory networks involving both the AraC and LacI inducible transcription factors

Manjunatha Kogenaru[1,2,3*] and Sander J. Tans[1]

[1]FOM Institute for Atomic and Molecular Physics, Science Park 104, 1098 XG Amsterdam, The Netherlands,

[2,3]Present address: [2]EMBL/CRG Systems Biology Research Unit, Centre for Genomic Regulation (CRG), Dr. Aiguader 88, 08003 Barcelona, Spain. [3]Universitat Pompeu Fabra (UPF), 08003 Barcelona, Spain.

[*]Corresponding author

Email addresses:

MK: mkogenaru@yahoo.com

SJT: tans@amolf.nl

1. Strain engineering

We selected a 40 base pairs (bps) regions from the 5` and 3` ends of the *lacI* as a homology arms and added this to the either ends of the chloramphenicol acetyltransferase (*cat*) selection cassette. This cassette is in turn flanked by the Cre recombinase recognition loxP sites[1]. This whole construct was PCR amplified as a single product and digested with 10 U of *DpnI* for 2 hours at 37 °C. This digested product was further purified by loading on to the agarose gel, before transforming into the parental BW27783 strain by electroporation. Prior to this, the BW27783 was transformed with the phage lambda-derived Red recombinase encoding helper plasmid pKD46[2] (GenBank™ Accession number AY048746). The transformants from the *lacI* deletion were selected on the LB agar plate containing 15 µg/ml Chloramphenicol. The resulting chloramphenicol-resistant clones were screened for the positive recombinants by colony PCR. The confirmed positive clone was further colony-purified once under non-selective condition at 37 °C, and subsequently tested for Ampicillin sensitivity to confirm the loss of the helper plasmid. This clone was further verified for the desired mutation.

V2LacI-del_fr
GTGAAACCAGTAACGTTATACGATGTCGCAGAGTATGCCGAGGAAGCGGAATTCCGGACC

V2LacI-del_rv
TCACTGCCCGCTTTCCAGTCGGGAAACCTGTCGTGCCAGCTACCGTCGACGGATCCCCGG

lacI

```
>gi|1657477|gb|U73857.1|ECU73857 Escherichia coli str. K-12 substr. MG1655
GAACAACGGGTGATTGGCTGTCTGAATCTGGTGTATATGGCGAGCGCAATGACCATTGAACAGGCAGCGGAAAAGCATCT
TCCGGCGCTACAACGGGTAGCAAAACAGATCGAAGAAGGGGTTGAATCGCAGGCTATTCTGGTGGCCGGAAGGCGAAGCG
GCATGCATTTACGTTGACACCATCGAATGGCGCAAAACCTTTCGCGGTATGGCATGATAGCGCCCGGAAGAGAGTCAATT
CAGGGTGGTGAATGTGAAACCAGTAACGTTATACGATGTCGCAGAGTATGCCGGTGTCTCTTATCAGACCGTTTCCCGCG
TGGTGAACCAGGCCAGCCACGTTTCTGCGAAAACGCGGGAAAAAGTGGAAGCGGCGATGGCGGAGCTGAATTACATTCCC
AACCGCGTGGCACAACAACTGGCGGGCAAACAGTCGTTGCTGATTGGCGTTGCCACCTCCAGTCTGGCCCTGCACGCGCC
GTCGCAAATTGTCGCGGCGATTAAATCTCGCGCCGATCAACTGGGTGCCAGCGTGGTGGTGTCGATGGTAGAACGAAGCG
GCGTCGAAGCCTGTAAAGCGGCGGTGCACAATCTTCTCGCGCAACGCGTCAGTGGGCTGATCATTAACTATCCGCTGGAT
GACCAGGATGCCATTGCTGTGGAAGCTGCCTGCACTAATGTTCCGGCGTTATTTCTTGATGTCTCTGACCAGACACCCAT
CAACAGTATTATTTTCTCCCATGAAGACGGTACGCGACTGGGCGTGGAGCATCTGGTCGCATTGGGTCACCAGCAAATCG
CGCTGTTAGCGGGCCCATTAAGTTCTGTCTCGGCGCGTCTGCGTCTGGCTGGCTGGCATAAATATCTCACTCGCAATCAA
ATTCAGCCGATAGCGGAACGGGAAGGCGACTGGAGTGCCATGTCCGGTTTTCAACAAACCATGCAAATGCTGAATGAGGG
CATCGTTCCCACTGCGATGCTGGTTGCCAACGATCAGATGGCGCTGGGCGCAATGCGCGCCATTACCGAGTCCGGGCTGC
GCGTTGGTGCGGATATCTCGGTAGTGGGATACGACGATACCGAAGACAGCTCATGTTATATCCCGCCGTCAACCACCATC
AAACAGGATTTTCGCCTGCTGGGGCAAACCAGCGTGGACCGCTTGCTGCAACTCTCTCAGGGCCAGGCGGTGAAGGGCAA
TCAGCTGTTGCCCGTCTCACTGGTGAAAAGAAAAACCACCCTGGCGCCCAATACGCAAACCGCCTCTCCCCGCGCGTTGG
CCGATTCATTAATGCAGCTGGCACGACAGGTTTCCCGACTGGAAAGCGGGCAGTGAGCGCAACGCAATTAATGTGAGTTA
GC
```

lacI homology arm sequence
loxP sites
Chloramphenicol acetyltransferase antibiotic resistance gene

LacI-del-test_rv
ACGTAAGAGGTTCCAACTTTCACC

LacI-del-test_fr
CCCGCCCTGCCACTCATCGCAGTAC

LacIdel-test2_fr
CGCAGGCTATTCTGGTGGCC

LacIdel-test2_rv
TCACTGCCCGCTTTCCAGTCG

```
>MK01 sequence at engineered locus
GAACAACGGGTGATTGGCTGTCTGAATCTGGTGTATATGGCGAGCGCAATGACCATTGAACAGGCAGCGGAAAAGCATCT
TCCGGCGCTACAACGGGTAGCAAAACAGATCGAAGAAGGGGTTGAATCGCAGGCTATTCTGGTGGCCGGAAGGCGAAGCG
GCATGCATTTACGTTGACACCATCGAATGGCGCAAAACCTTTCGCGGTATGGCATGATAGCGCCCGGAAGAGAGTCAATT
CAGGGTGGTGAATGTGAAACCAGTAACGTTATACGATGTCGCAGAGTATGCCGAGGAAGCGGAATTCCGGACCGGCGCGC
CAAAAAAACCCGCCGAAGCGGGTTTTTTTCCCGGGATAACTTCGTATAATGTATGCTATACGAAGTTATCGATTACGCCC
CGCCCTGCCACTCATCGCAGTACTGTTGTAATTCATTAAGCATTCTGCCGACATGGAAGCCATCACAAACGGCATGATGA
ACCTGAATCGCCAGCGGCATCAGCACCTTGTCGCCTTGCGTATAATATTTGCCCATGGTGAAAACGGGGGCGAAGAAGTT
GTCCATATTGGCCACGTTTAAATCAAAACTGGTGAAACTCACCCAGGGATTGGCTGAGACGAAAAACATATTCTCAATAA
ACCCTTTAGGGAAATAGGCCAGGTTTTCACCGTAACACGCCACATCTTGCGAATATATGTGTAGAAACTGCCGGAAATCG
TCGTGGTATTCACTCCAGAGCGATGAAAACGTTTCAGTTTGCTCATGGAAAACGGTGTAACAAGGGTGAACACTATCCCA
TATCACCAGCTCACCGTCTTTCATTGCCATACGGAATTCCGGATGAGCATTCATCAGGCGGGCAAGAATGTGAATAAAGG
CCGGATAAAACTTGTGCTTATTTTTCTTTACGGTCTTTAAAAAGGCCGTAATATCCAGCTGAACGGTCTGGTTATAGGTA
CATTGAGCAACTGACTGAAATGCCTCAAAATGTTCTTTACGATGCCATTGGGATATATCAACGGTGGTATATCCAGTGAT
TTTTTTCTCCATTTTAGCTTCCTTAGCTCCTGAAAATCTCGATAACTCAAAAAATACGCCCGGTAGTGATCTTATTTCAT
TATGGTGAAAGTTGGAACCTCTTACGTGCCAATCGATAACTTCGTATAATGTATGCTATACGAAGTTATCCCGGGGATCC
GTCGACGGTAGCTGGCACGACAGGTTTCCCGACTGGAAAGCGGGCAGTGA
```

2. DNA constructs

2.1 Construction of the plasmids used in the competition assay

The gene *lacI* along with the promoter *lacI*^{Q} were amplified from pET-28b+ vector (Novagen) as a template. The *eYFP* gene was amplified from the in-house plasmid pLacImCherry-eYFP (M.K. and

S.J.T, unpublished) as a template. Using these two PCR products as a template, an overlap PCR was performed. The resulting PCR product was cloned onto the vector backbone of pLacImCherry-eYFP with XbaI and SalI restriction enzymes (Figure S2a). Similarly, *eYFP* was replaced with *mCherry* to obtain the construct that constitutively express (Figure S2b).

2.2 Construction of the plasmids used in the simultaneous induction assay

The *araC-P*$_{BAD}$ part was PCR amplified from the in-house plasmid that is similar to the pBAD24 cloning vector (GenBank™ Accession number X81837). The resulting PCR product was cloned into pINV-110[3] plasmid backbone using AatII and XhoI restriction enzymes, to obtain an intermediate plasmid, pAraC. The *mCherry* sequence was cloned into the pAraC using EcoRI and XhoI restriction enzymes (Figure S3a).

The gene *lacI* along with its promoter was cloned into the vector backbone of pLacImCherry-eYFP using HindIII and SalI restriction enzymes (Figure S3b).

Figure S1

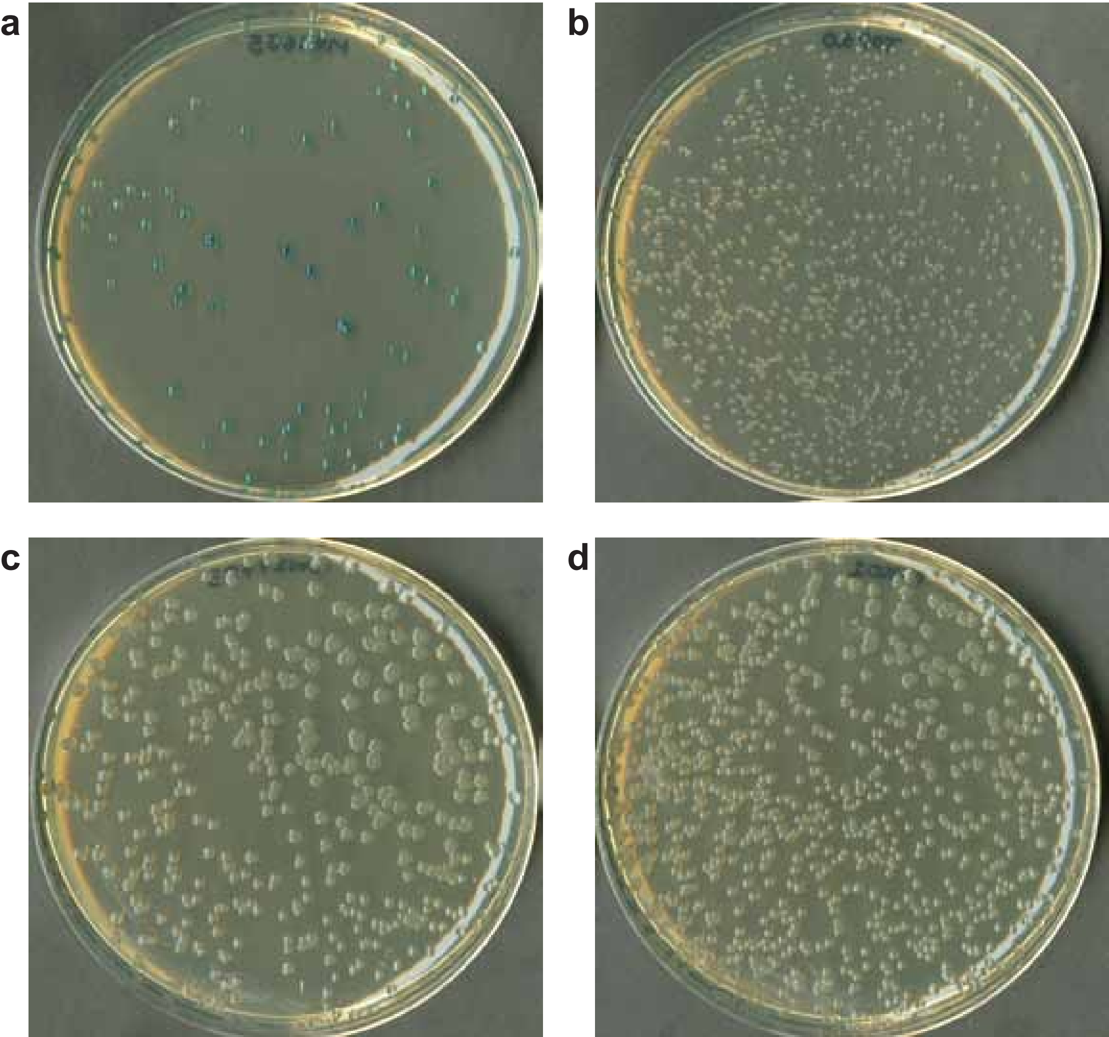

Figure S2a

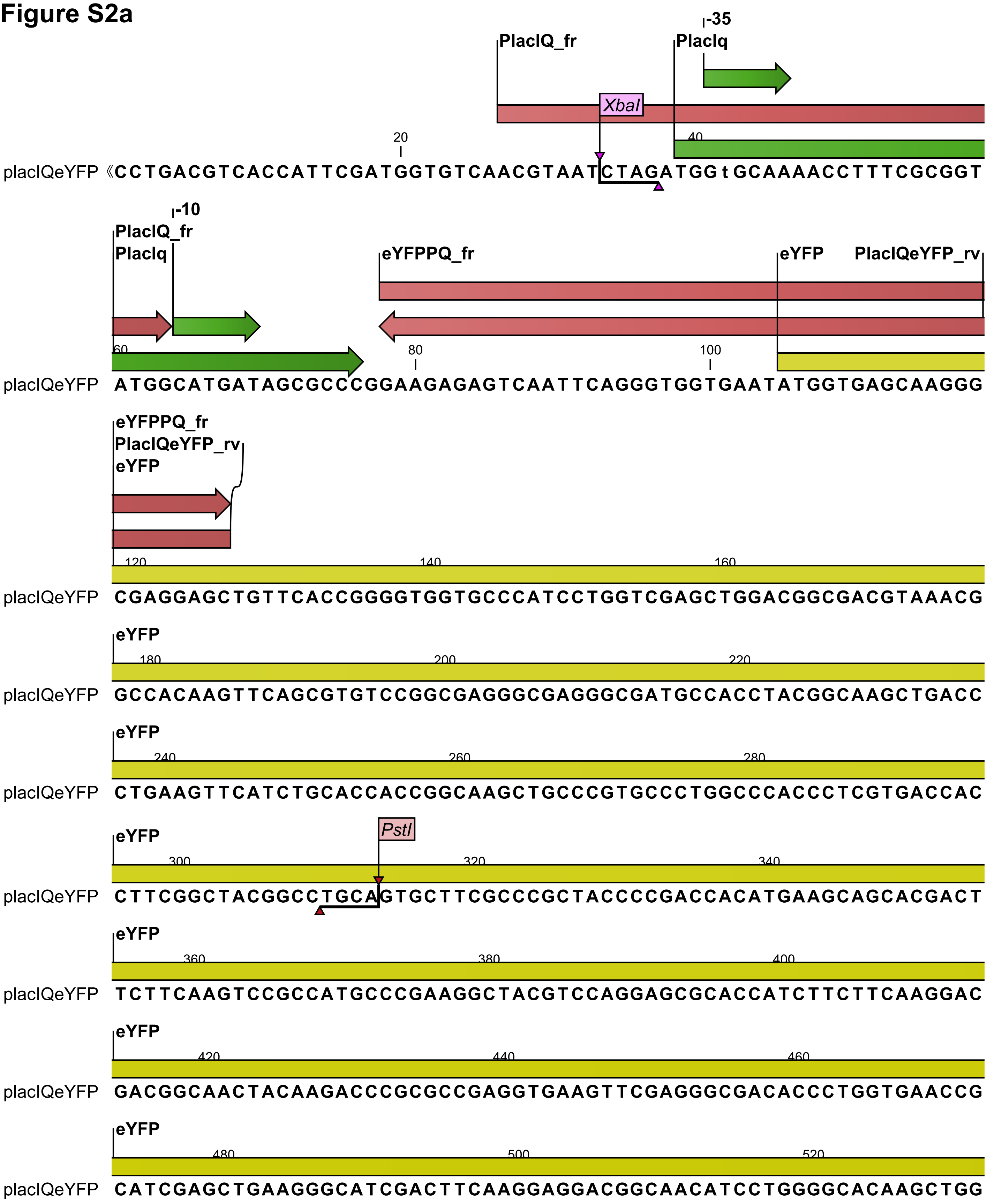

-35
PlacIQ_fr
PlacIq
XbaI
20
40
placIQeYFP 《CCTGACGTCACCATTCGATGGTGTCAACGTAATCTAGATGGtGCAAAACCTTTCGCGGT
-10
PlacIQ_fr
PlacIq
eYFPPQ_fr
eYFP
PlacIQeYFP_rv
60
80
100
placIQeYFP ATGGCATGATAGCGCCCGGAAGAGAGTCAATTCAGGGTGGTGAATATGGTGAGCAAGGG
eYFPPQ_fr
PlacIQeYFP_rv
eYFP
120
140
160
placIQeYFP CGAGGAGCTGTTCACCGGGGTGGTGCCCATCCTGGTCGAGCTGGACGGCGACGTAAACG
eYFP
180
200
220
placIQeYFP GCCACAAGTTCAGCGTGTCCGGCGAGGGCGAGGGCGATGCCACCTACGGCAAGCTGACC
eYFP
240
260
280
placIQeYFP CTGAAGTTCATCTGCACCACCGGCAAGCTGCCCGTGCCCTGGCCCACCCTCGTGACCAC
eYFP
PstI
300
320
340
placIQeYFP CTTCGGCTACGGCCTGCAGTGCTTCGCCCGCTACCCCGACCACATGAAGCAGCACGACT
eYFP
360
380
400
placIQeYFP TCTTCAAGTCCGCCATGCCCGAAGGCTACGTCCAGGAGCGCACCATCTTCTTCAAGGAC
eYFP
420
440
460
placIQeYFP GACGGCAACTACAAGACCCGCGCCGAGGTGAAGTTCGAGGGCGACACCCTGGTGAACCG
eYFP
480
500
520
placIQeYFP CATCGAGCTGAAGGGCATCGACTTCAAGGAGGACGGCAACATCCTGGGGCACAAGCTGG

eYFP

540 560 580

placIQeYFP AGTACAACTACAACAGCCACAACGTCTATATCATGGCCGACAAGCAGAAGAACGGCATC

eYFP

600 620 640

placIQeYFP AAGGTGAACTTCAAGATCCGCCACAACATCGAGGACGGCAGCGTGCAGCTCGCCGACCA

eYFP

660 680 700

placIQeYFP CTACCAGCAGAACACCCCCATCGGCGACGGCCCCGTGCTGCTGCCCGACAACCACTACC

eYFP

720 740 760

placIQeYFP TGAGCTACCAGTCCGCCCTGAGCAAAGACCCCAACGAGAAGCGCGATCACATGGTCCTG

eYFP

SalI-eYFP_rv

780 800 820

placIQeYFP CTGGAGTTCGTGACCGCCGCCGGGATCACTCTCGGCATGGACGAGCTGTACAAGTAACC

SalI-eYFP_rv

T1 Term

SalI

840 860 880

placIQeYFP TAGGGGAGTCGACCCGCTGCATCAAATAAAACGAAAGGCTCAGTCGAAAGACTGGGCCT

T1 Term

900 920 940

placIQeYFP TTCGTTTTATCTGTTGTTTGTCGGTGAACGCTCTCCTGAGTAGGACAAATCCGCCGCCC

T1 Term

XhoI

960 980 1,000

placIQeYFP TAGACTCGGCCGTCTCGAGGTCTTCGGTTTCCGTGTTTCGTAAAGTCTGGAAACGCGGA

1,020 1,040 1,060

Figure S2b

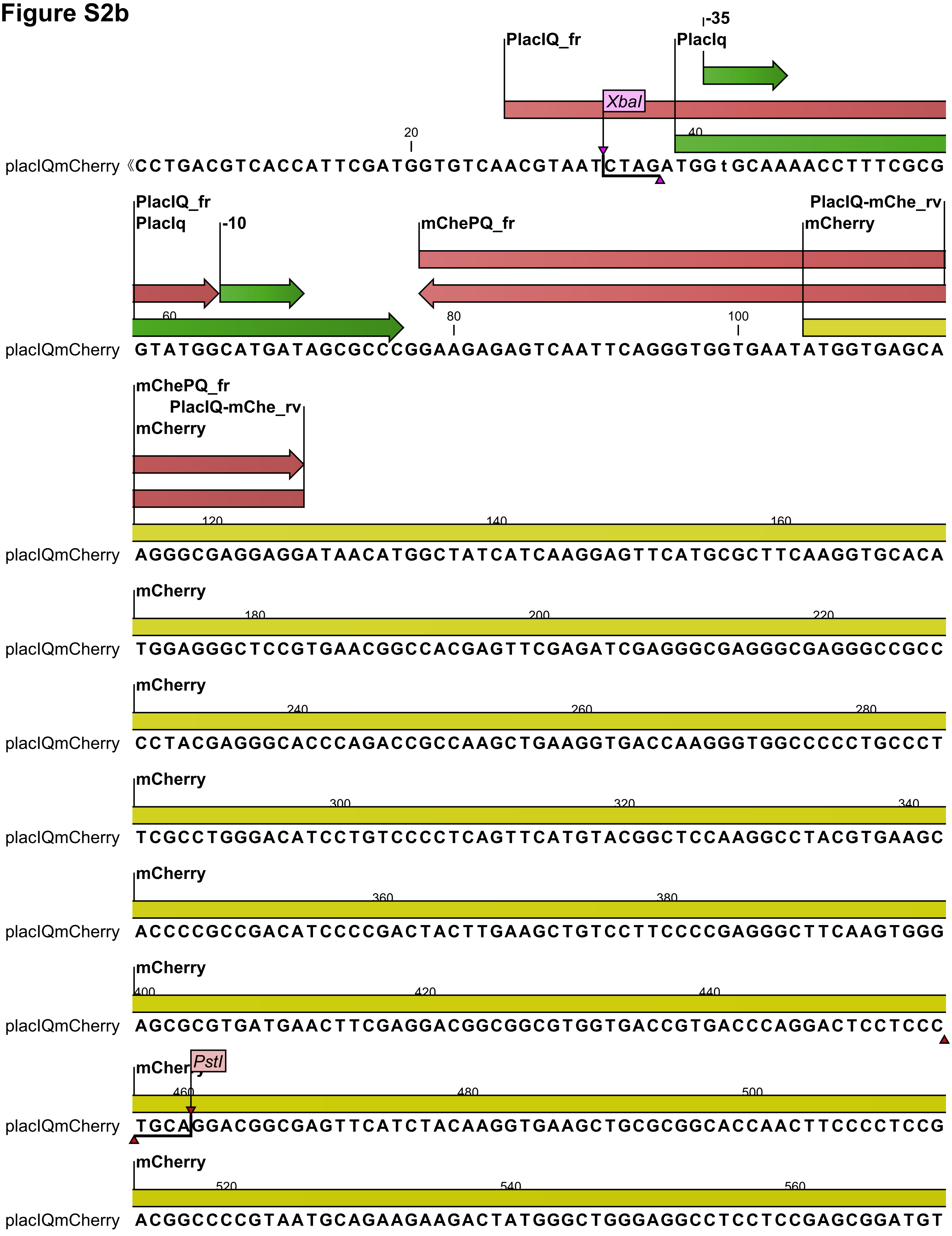

-35
PlacIQ_fr
PlacIq
XbaI
20
40
placIQmCherry CCTGACGTCACCATTCGATGGTGTCAACGTAATCTAGATGGtGCAAAACCTTTCGCG
PlacIQ_fr
PlacIq
-10
mChePQ_fr
PlacIQ-mChe_rv
mCherry
60
80
100
placIQmCherry GTATGGCATGATAGCGCCCGGAAGAGAGTCAATTCAGGGTGGTGAATATGGTGAGCA
mChePQ_fr
PlacIQ-mChe_rv
mCherry
120
140
160
placIQmCherry AGGGCGAGGAGGATAACATGGCTATCATCAAGGAGTTCATGCGCTTCAAGGTGCACA
mCherry
180
200
220
placIQmCherry TGGAGGGCTCCGTGAACGGCCACGAGTTCGAGATCGAGGGCGAGGGCGAGGGCCGCC
mCherry
240
260
280
placIQmCherry CCTACGAGGGCACCCAGACCGCCAAGCTGAAGGTGACCAAGGGTGGCCCCCTGCCCT
mCherry
300
320
340
placIQmCherry TCGCCTGGGACATCCTGTCCCCTCAGTTCATGTACGGCTCCAAGGCCTACGTGAAGC
mCherry
360
380
placIQmCherry ACCCCGCCGACATCCCCGACTACTTGAAGCTGTCCTTCCCCGAGGGCTTCAAGTGGG
mCherry
400
420
440
placIQmCherry AGCGCGTGATGAACTTCGAGGACGGCGGCGTGGTGACCGTGACCCAGGACTCCTCCC
mCherry
PstI
460
480
500
placIQmCherry TGCAGGACGGCGAGTTCATCTACAAGGTGAAGCTGCGCGGCACCAACTTCCCCTCCG
mCherry
520
540
560
placIQmCherry ACGGCCCCGTAATGCAGAAGAAGACTATGGGCTGGGAGGCCTCCTCCGAGCGGATGT

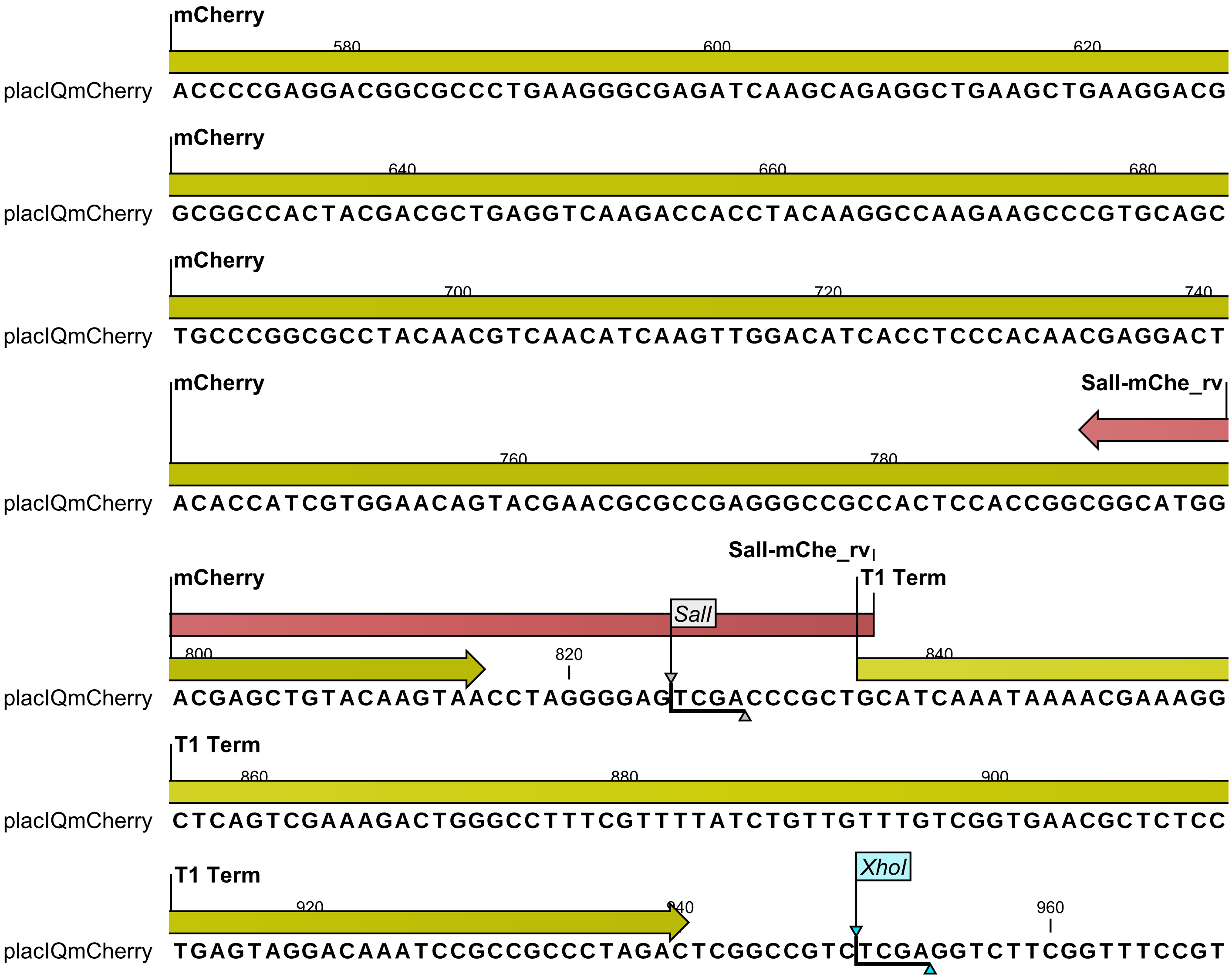
mCherry
580
600
620
placIQmCherry ACCCCGAGGACGGCGCCCTGAAGGGCGAGATCAAGCAGAGGCTGAAGCTGAAGGACG
mCherry
640
660
680
placIQmCherry GCGGCCACTACGACGCTGAGGTCAAGACCACCTACAAGGCCAAGAAGCCCGTGCAGC
mCherry
700
720
740
placIQmCherry TGCCCGGCGCCTACAACGTCAACATCAAGTTGGACATCACCTCCCACAACGAGGACT
mCherry
SalI-mChe_rv
760
780
placIQmCherry ACACCATCGTGGAACAGTACGAACGCGCCGAGGGCCGCCACTCCACCGGCGGCATGG
SalI-mChe_rv
mCherry
T1 Term
SalI
800
820
840
placIQmCherry ACGAGCTGTACAAGTAACCTAGGGGAGTCGACCCGCTGCATCAAATAAAACGAAAGG
T1 Term
860
880
900
placIQmCherry CTCAGTCGAAAGACTGGGCCTTTCGTTTTATCTGTTGTTTGTCGGTGAACGCTCTCC
T1 Term
XhoI
920
940
960
placIQmCherry TGAGTAGGACAAATCCGCCGCCCTAGACTCGGCCGTCTCGAGGTCTTCGGTTTCCGT

Figure S3a

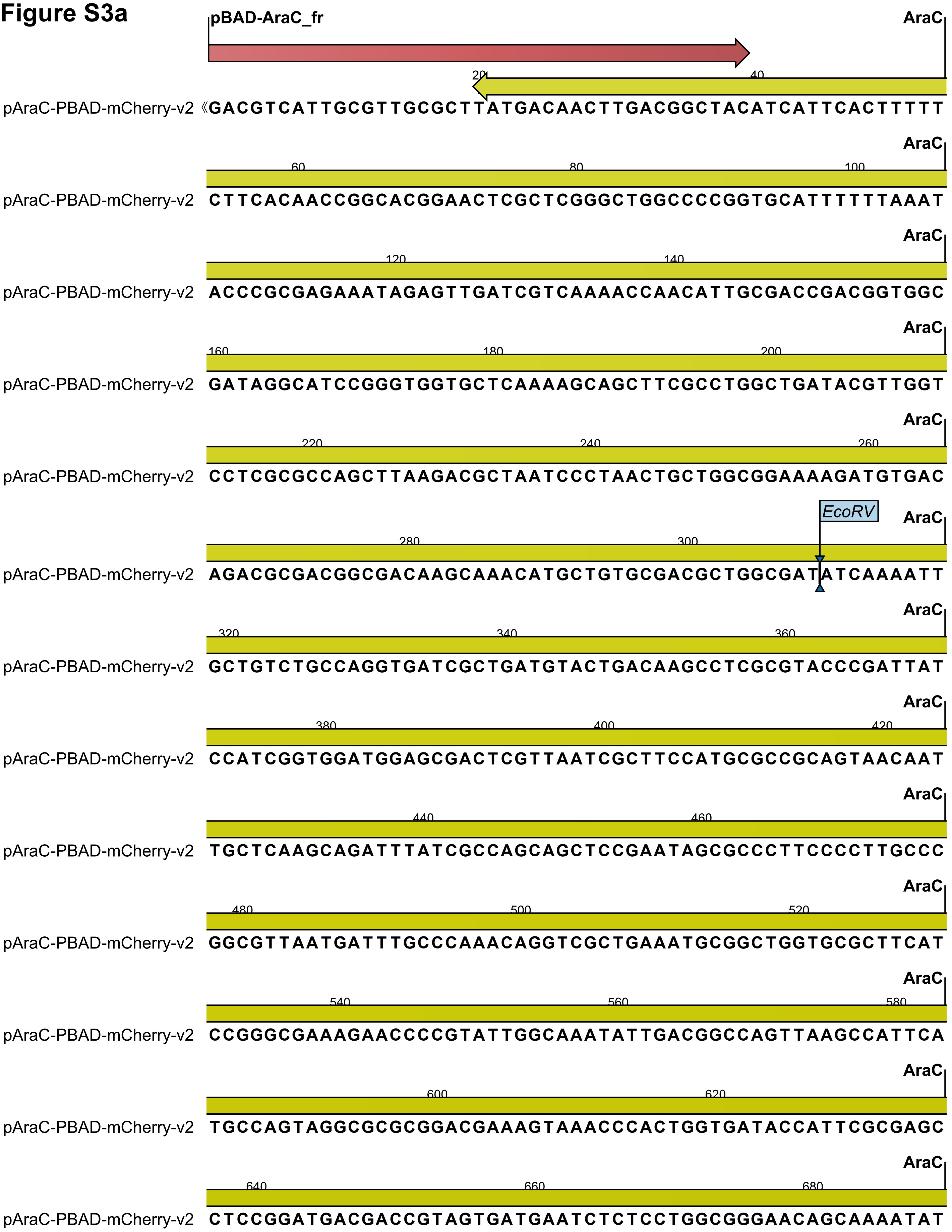

pBAD-AraC_fr
AraC
EcoRV
pAraC-PBAD-mCherry-v2 GACGTCATTGCGTTGCGCTTATGACAACTTGACGGCTACATCATTCACTTTTT
pAraC-PBAD-mCherry-v2 CTTCACAACCGGCACGGAACTCGCTCGGGCTGGCCCCGGTGCATTTTTTAAAT
pAraC-PBAD-mCherry-v2 ACCCGCGAGAAATAGAGTTGATCGTCAAAACCAACATTGCGACCGACGGTGGC
pAraC-PBAD-mCherry-v2 GATAGGCATCCGGGTGGTGCTCAAAAGCAGCTTCGCCTGGCTGATACGTTGGT
pAraC-PBAD-mCherry-v2 CCTCGCGCCAGCTTAAGACGCTAATCCCTAACTGCTGGCGGAAAAGATGTGAC
pAraC-PBAD-mCherry-v2 AGACGCGACGGCGACAAGCAAACATGCTGTGCGACGCTGGCGATATCAAAATT
pAraC-PBAD-mCherry-v2 GCTGTCTGCCAGGTGATCGCTGATGTACTGACAAGCCTCGCGTACCCGATTAT
pAraC-PBAD-mCherry-v2 CCATCGGTGGATGGAGCGACTCGTTAATCGCTTCCATGCGCCGCAGTAACAAT
pAraC-PBAD-mCherry-v2 TGCTCAAGCAGATTTATCGCCAGCAGCTCCGAATAGCGCCCTTCCCCTTGCCC
pAraC-PBAD-mCherry-v2 GGCGTTAATGATTTGCCCAAACAGGTCGCTGAAATGCGGCTGGTGCGCTTCAT
pAraC-PBAD-mCherry-v2 CCGGGCGAAAGAACCCCGTATTGGCAAATATTGACGGCCAGTTAAGCCATTCA
pAraC-PBAD-mCherry-v2 TGCCAGTAGGCGCGCGGACGAAAGTAAACCCACTGGTGATACCATTCGCGAGC
pAraC-PBAD-mCherry-v2 CTCCGGATGACGACCGTAGTGATGAATCTCTCCTGGCGGGAACAGCAAAATAT

AraC
700 720 740
pAraC-PBAD-mCherry-v2 CACCCGGTCGGCAAACAAATTCTCGTCCCTGATTTTTCACCACCCCCTGACCG

AraC
760 780
pAraC-PBAD-mCherry-v2 CGAATGGTGAGATTGAGAATATAACCTTTCATTCCCAGCGGTCGGTCGATAAA

AraC
800 820 840
pAraC-PBAD-mCherry-v2 AAAATCGAGATAACCGTTGGCCTCAATCGGCGTTAAACCCGCCACCAGATGGG

AraC
860 880 900
pAraC-PBAD-mCherry-v2 CATTAAACGAGTATCCCGGCAGCAGGGGATCATTTTGCGCTTCAGCCATACTT

AraC P_BAD
920 940
pAraC-PBAD-mCherry-v2 TTCATACTCCCGCCATTCAGAGAAGAAACCAATTGTCCATATTGCATCAGACA

P_BAD AgeI
960 980 1 000
pAraC-PBAD-mCherry-v2 TTGCCGTCACTGCGTCTTTTACTGGCTCTTCTCGCTAACCAAACCGGTAACCC

P_BAD
1 020 1 040 1 060
pAraC-PBAD-mCherry-v2 CGCTTATTAAAAGCATTCTGTAACAAAGCGGGACCAAAGCCATGACAAAAACG

P_BAD pbadO1
1 080 1 100
pAraC-PBAD-mCherry-v2 CGTAACAAAAGTGTCTATAATCACGGCAGAAAAGTCCACATTGATTATTTGCA

P_BAD CAP bs I2-I1-Region BamHI
1 120 1 140 1 160
pAraC-PBAD-mCherry-v2 CGGCGTCACACTTTGCTATGCCATAGCATTTTTATCCATAAGATTAGCGGATC

-35
I2-I1-Region
P_BAD -10
araBAD_fr pBAD-AraC_rv
1 180 1 200
pAraC-PBAD-mCherry-v2 CTACCTGACGCTTTTTATCGCAACTCTCTACTGTTTCTCCATACCCGTTTTTT

P_BAD RBS pBAD-AraC_rv EcoRI-mChe_fr mCherry EcoRI

1,220 1,240 1,260

pAraC-PBAD-mCherry-v2 GGGCTAACAGGAGGAAGAATTCATGGTGAGCAAGGGCGAGGAGGATAACATGG

mCherry 1,280 1,300 1,320

pAraC-PBAD-mCherry-v2 CTATCATCAAGGAGTTCATGCGCTTCAAGGTGCACATGGAGGGCTCCGTGAAC

mCherry 1,340 1,360

pAraC-PBAD-mCherry-v2 GGCCACGAGTTCGAGATCGAGGGCGAGGGCGAGGGCCGCCCCTACGAGGGCAC

mCherry 1,380 1,400 1,420

pAraC-PBAD-mCherry-v2 CCAGACCGCCAAGCTGAAGGTGACCAAGGGTGGCCCCCTGCCCTTCGCCTGGG

mCherry 1,440 1,460 1,480

pAraC-PBAD-mCherry-v2 ACATCCTGTCCCCTCAGTTCATGTACGGCTCCAAGGCCTACGTGAAGCACCCC

mCherry 1,500 1,520

pAraC-PBAD-mCherry-v2 GCCGACATCCCCGACTACTTGAAGCTGTCCTTCCCCGAGGGCTTCAAGTGGGA

mCherry 1,540 1,560 1,580

pAraC-PBAD-mCherry-v2 GCGCGTGATGAACTTCGAGGACGGCGGCGTGGTGACCGTGACCCAGGACTCCT

mCherry PstI 1,600 1,620 1,640

pAraC-PBAD-mCherry-v2 CCCTGCAGGACGGCGAGTTCATCTACAAGGTGAAGCTGCGCGGCACCAACTTC

mCherry 1,660 1,680

pAraC-PBAD-mCherry-v2 CCCTCCGACGGCCCCGTAATGCAGAAGAAGACTATGGGCTGGGAGGCCTCCTC

mCherry 1,700 1,720 1,740

pAraC-PBAD-mCherry-v2 CGAGCGGATGTACCCCGAGGACGGCGCCCTGAAGGGCGAGATCAAGCAGAGGC

mCherry 1,760 1,780 1,800

pAraC-PBAD-mCherry-v2 TGAAGCTGAAGGACGGCGGCCACTACGACGCTGAGGTCAAGACCACCTACAAG

mCherry 1,820 1,840

pAraC-PBAD-mCherry-v2 GCCAAGAAGCCCGTGCAGCTGCCCGGCGCCTACAACGTCAACATCAAGTTGGA

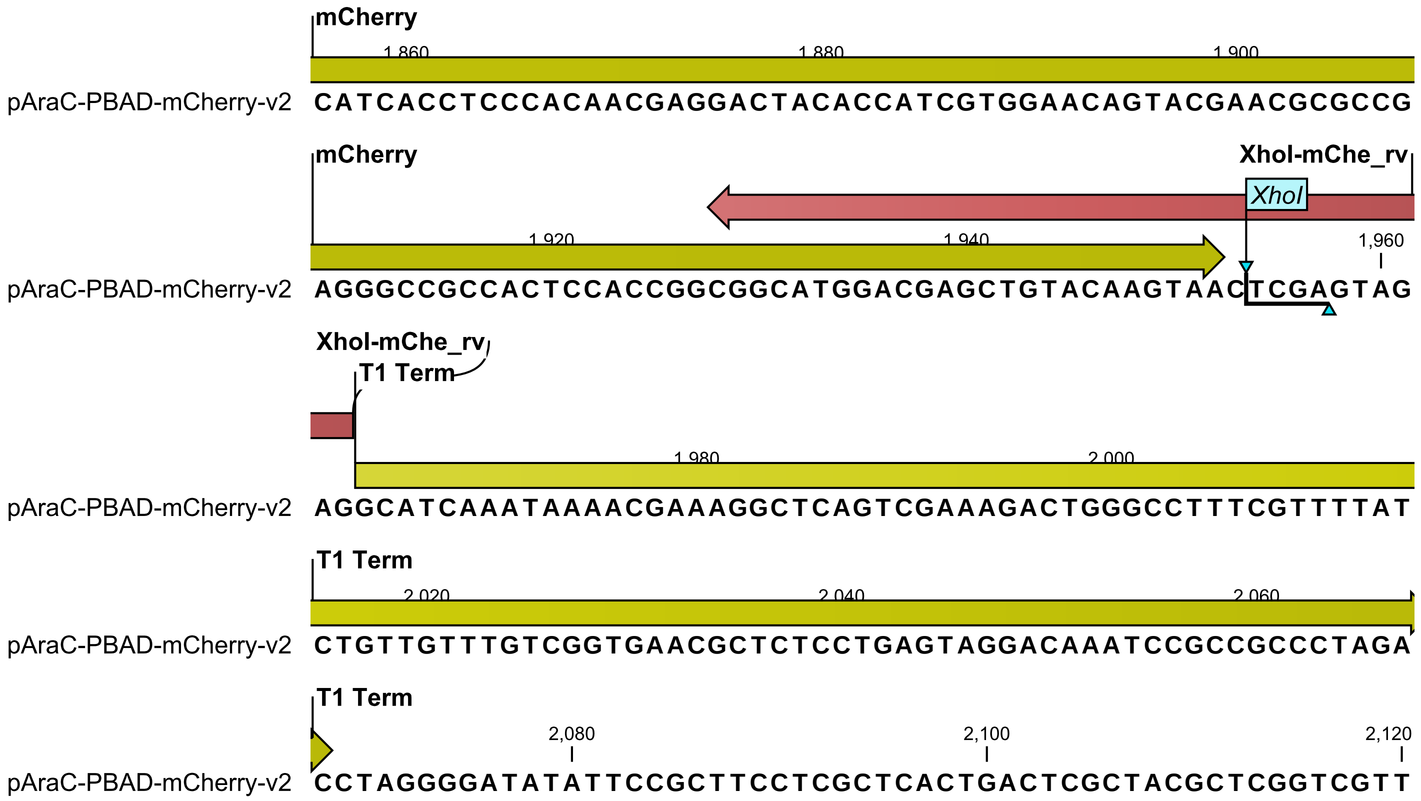
mCherry
1,860
1,880
1,900
pAraC-PBAD-mCherry-v2 CATCACCTCCCACAACGAGGACTACACCATCGTGGAACAGTACGAACGCGCCG
mCherry
XhoI-mChe_rv
XhoI
1,920
1,940
1,960
pAraC-PBAD-mCherry-v2 AGGGCCGCCACTCCACCGGCGGCATGGACGAGCTGTACAAGTAACTCGAGTAG
XhoI-mChe_rv
T1 Term
1,980
2,000
pAraC-PBAD-mCherry-v2 AGGCATCAAATAAAACGAAAGGCTCAGTCGAAAGACTGGGCCTTTCGTTTTAT
T1 Term
2,020
2,040
2,060
pAraC-PBAD-mCherry-v2 CTGTTGTTTGTCGGTGAACGCTCTCCTGAGTAGGACAAATCCGCCGCCCTAGA
T1 Term
2,080
2,100
2,120
pAraC-PBAD-mCherry-v2 CCTAGGGGATATATTCCGCTTCCTCGCTCACTGACTCGCTACGCTCGGTCGTT

Figure S3b

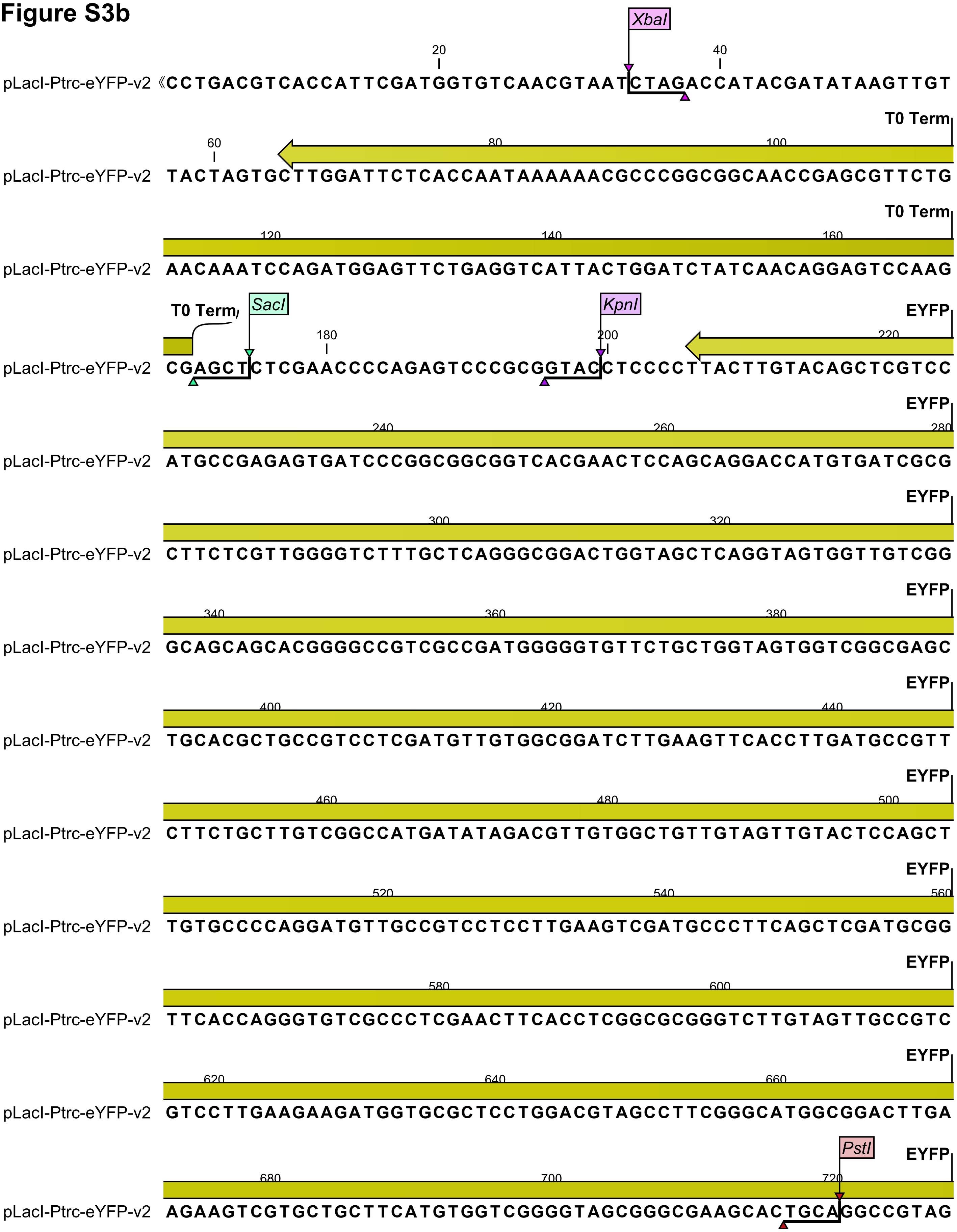

XbaI
pLacI-Ptrc-eYFP-v2 《CCTGACGTCACCATTCGATGGTGTCAACGTAATCTAGACCATACGATATAAGTTGT
T0 Term
pLacI-Ptrc-eYFP-v2 TACTAGTGCTTGGATTCTCACCAATAAAAACGCCCGGCGGCAACCGAGCGTTCTG
T0 Term
pLacI-Ptrc-eYFP-v2 AACAAATCCAGATGGAGTTCTGAGGTCATTACTGGATCTATCAACAGGAGTCCAAG
T0 Term
SacI
KpnI
EYFP
pLacI-Ptrc-eYFP-v2 CGAGCTCTCGAACCCCAGAGTCCCGCGGTACCTCCCCTTACTTGTACAGCTCGTCC
EYFP
pLacI-Ptrc-eYFP-v2 ATGCCGAGAGTGATCCCGGCGGCGGTCACGAACTCCAGCAGGACCATGTGATCGCG
EYFP
pLacI-Ptrc-eYFP-v2 CTTCTCGTTGGGGTCTTTGCTCAGGGCGGACTGGTAGCTCAGGTAGTGGTTGTCGG
EYFP
pLacI-Ptrc-eYFP-v2 GCAGCAGCACGGGGCCGTCGCCGATGGGGGTGTTCTGCTGGTAGTGGTCGGCGAGC
EYFP
pLacI-Ptrc-eYFP-v2 TGCACGCTGCCGTCCTCGATGTTGTGGCGGATCTTGAAGTTCACCTTGATGCCGTT
EYFP
pLacI-Ptrc-eYFP-v2 CTTCTGCTTGTCGGCCATGATATAGACGTTGTGGCTGTTGTAGTTGTACTCCAGCT
EYFP
pLacI-Ptrc-eYFP-v2 TGTGCCCCAGGATGTTGCCGTCCTCCTTGAAGTCGATGCCCTTCAGCTCGATGCGG
EYFP
pLacI-Ptrc-eYFP-v2 TTCACCAGGGTGTCGCCCTCGAACTTCACCTCGGCGCGGGTCTTGTAGTTGCCGTC
EYFP
pLacI-Ptrc-eYFP-v2 GTCCTTGAAGAAGATGGTGCGCTCCTGGACGTAGCCTTCGGGCATGGCGGACTTGA
PstI
EYFP
pLacI-Ptrc-eYFP-v2 AGAAGTCGTGCTGCTTCATGTGGTCGGGGTAGCGGGCGAAGCACTGCAGGCCGTAG

EYFP

740 760 780

pLacI-Ptrc-eYFP-v2 CCGAAGGTGGTCACGAGGGTGGGCCAGGGCACGGGCAGCTTGCCGGTGGTGCAGAT

EYFP

800 820 840

pLacI-Ptrc-eYFP-v2 GAACTTCAGGGTCAGCTTGCCGTAGGTGGCATCGCCCTCGCCCTCGCCGGACACGC

EYFP

860 880

pLacI-Ptrc-eYFP-v2 TGAACTTGTGGCCGTTTACGTCGCCGTCCAGCTCGACCAGGATGGGCACCACCCCG

EYFP *BglII* RBS

900 920 940

pLacI-Ptrc-eYFP-v2 GTGAACAGCTCCTCGCCCTTGCTCACCATAGATCTTTCCTGCCATTCGTGAATTGT

-35

-10 Ptrc-mod-(no O3)

960 980 1,000

pLacI-Ptrc-eYFP-v2 TATCCGCTCACAATTCCACACATTATACGAGCCGGATGATTAATTGTCAACAGCTC

Ptrc-mod-(no O3)

1,020 1,040 1,060

pLacI-Ptrc-eYFP-v2 ATTTCAGAATATTTGCCAGAACCGTTATGATGTCGGCGCAAAAAACATTATCCAGA

Ptrc-mod-(no O3)

1,080 1,100 1,120

pLacI-Ptrc-eYFP-v2 ACGGGAGTGCGCCTTGAGCGACACGAATTATGCAGTGATTTACGACCTGCACAGCC

Ptrc-mod-(no O3)

1,140 1,160

pLacI-Ptrc-eYFP-v2 ATACCACAGCTTCCGATGGCTGCCTGACGCCAGAAGCATTGGTGCACCGTGCAGTC

Ptrc-mod-(no O3)

1,180 1,200 1,220

pLacI-Ptrc-eYFP-v2 GATGATAAGCTGTCAAACCAGATCAATTCGCGCTAACTCACATTAATTCACTCCAA

1,240 1,260 1,280

pLacI-Ptrc-eYFP-v2 GGTGAACCTTAGTGTGCACGAGGATAAGAACCGGGTGCCGTACGTCAAGGGCGCTA

1,300 1,320 1,340

pLacI-Ptrc-eYFP-v2 CGGAACGGTTCGTCTCGTCGCCGGAGGATGTTTTCGAGGTGATCGAGGAGGGCAAA

1,360 1,380 1,400

pLacI-Ptrc-eYFP-v2 TCCAATCGTCACATCGCTGTGACAAACATGAACGAGCATTCTTCGCGATCCCACTC

1,420 1,440

pLacI-Ptrc-eYFP-v2 AGTATTCCTTATCAATGTGAAGCAGGAGAACCTGGAGAACCAGAAGAAACTATCCG

lacI_fr PlacI HindIII

1,460 1,480 1,500

pLacI-Ptrc-eYFP-v2 GCAAACTCTACCTGGTGGATTTGGCACAATTGGCGAATTGTCGAGGGAAAGCTTTG

-35
lacI_fr
PlacI
-10

1,520 1,540 1,560

pLacI-Ptrc-eYFP-v2 GCGCAAAACCTTTCGCGGTATGGCATGATAGCGCCCGGAAGAGAGTCAATTCAGGG

lacI
1,580 1,600 1,620

pLacI-Ptrc-eYFP-v2 TGGTGAATGTGAAACCAGTAACGTTATACGATGTCGCAGAGTATGCCGGTGTCTCT

lacI
1,640 1,660 1,680

pLacI-Ptrc-eYFP-v2 TATCAGACCGTTTCCCGCGTGGTGAACCAGGCCAGCCACGTTTCTGCGAAAACGCG

lacI
1,700 1,720

pLacI-Ptrc-eYFP-v2 GGAAAAAGTGGAAGCGGCGATGGCGGAGCTGAATTACATTCCCAACCGCGTGGCAC

lacI
1,740 1,760 1,780

pLacI-Ptrc-eYFP-v2 AACAACTGGCGGGCAAACAGTCGTTGCTGATTGGCGTTGCCACCTCCAGTCTGGCC

lacI
1,800 1,820 1,840

pLacI-Ptrc-eYFP-v2 CTGCACGCGCCGTCGCAAATTGTCGCGGCGATTAAATCTCGCGCCGATCAACTGGG

lacI
1,860 1,880 1,900

pLacI-Ptrc-eYFP-v2 TGCCAGCGTGGTGGTGTCGATGGTAGAACGAAGCGGCGTCGAAGCCTGTAAAGCGG

lacI
1,920 1,940 1,960

pLacI-Ptrc-eYFP-v2 CGGTGCACAATCTTCTCGCGCAACGCGTCAGTGGGCTGATCATTAACTATCCGCTG

lacI
1,980 2,000

pLacI-Ptrc-eYFP-v2 GATGACCAGGATGCCATTGCTGTGGAAGCTGCCTGCACTAATGTTCCGGCGTTATT

lacI
2,020 2,040 2,060

pLacI-Ptrc-eYFP-v2 TCTTGATGTCTCTGACCAGACACCCATCAACAGTATTATTTTCTCCCATGAAGACG

lacI
2,080 2,100 2,120

pLacI-Ptrc-eYFP-v2 GTACGCGACTGGGCGTGGAGCATCTGGTCGCATTGGGTCACCAGCAAATCGCGCTG

lacI

2,140 2,160 2,180

pLacI-Ptrc-eYFP-v2 TTAGCGGGCCCATTAAGTTCTGTCTCGGCGCGTCTGCGTCTGGCTGGCTGGCATAA

lacI

2,200 2,220 2,240

pLacI-Ptrc-eYFP-v2 ATATCTCACTCGCAATCAAATTCAGCCGATAGCGGAACGGGAAGGCGACTGGAGTG

lacI

2,260 2,280

pLacI-Ptrc-eYFP-v2 CCATGTCCGGTTTTCAACAAACCATGCAAATGCTGAATGAGGGCATCGTTCCCACT

lacI

2,300 2,320 2,340

pLacI-Ptrc-eYFP-v2 GCGATGCTGGTTGCCAACGATCAGATGGCGCTGGGCGCAATGCGCGCCATTACCGA

lacI EcoRV

2,360 2,380 2,400

pLacI-Ptrc-eYFP-v2 GTCCGGGCTGCGCGTTGGTGCGGATATCTCGGTAGTGGGATACGACGATACCGAAG

lacI

2,420 2,440 2,460

pLacI-Ptrc-eYFP-v2 ACAGCTCATGTTATATCCCGCCGTTAACCACCATCAAACAGGATTTTCGCCTGCTG

lacI

2,480 2,500 2,520

pLacI-Ptrc-eYFP-v2 GGGCAAACCAGCGTGGACCGCTTGCTGCAACTCTCTCAGGGCCAGGCGGTGAAGGG

lacI

2,540 2,560

pLacI-Ptrc-eYFP-v2 CAATCAGCTGTTGCCCGTCTCACTGGTGAAAAGAAAAACCACCCTGGCGCCCAATA

lacI

2,580 2,600 2,620

pLacI-Ptrc-eYFP-v2 CGCAAACCGCCTCTCCCCGCGCGTTGGCCGATTCATTAATGCAGCTGGCACGACAG

lacI_rv

lacI SalI T1 Term

2,640 2,660 2,680

pLacI-Ptrc-eYFP-v2 GTTTCCCGACTGGAAAGCGGGCAGTGACCTAGGGGAGTCGACCCGCTGCATCAAAT

T1 Term

2,700 2,720 2,740

pLacI-Ptrc-eYFP-v2 AAAACGAAAGGCTCAGTCGAAAGACTGGGCCTTTCGTTTTATCTGTTGTTTGTCGG

T1 Term XhoI

2,760 2,780 2,800

pLacI-Ptrc-eYFP-v2 TGAACGCTCTCCTGAGTAGGACAAATCCGCCGCCCTAGACTCGGCCGTCTCGAGGT

2,820 2,840

pLacI-Ptrc-eYFP-v2 CTTCGGTTTCCGTGTTTCGTAAAGTCTGGAAACGCGGAAGTCAGCGCCCTGCACCA

References

1. Watt RM, Wang J, Leong M, Kung HF, Cheah KS, Liu D, Danchin A, Huang JD: **Visualizing the proteome of Escherichia coli: an efficient and versatile method for labeling chromosomal coding DNA sequences (CDSs) with fluorescent protein genes.** *Nucleic Acids Res* 2007, **35:**e37.
2. Datsenko KA, Wanner BL: **One-step inactivation of chromosomal genes in Escherichia coli K-12 using PCR products.** *PNAS* 2000, **97:**6640-6645.
3. Yokobayashi Y, Weiss R, Arnold FH: **Directed evolution of a genetic circuit.** *PNAS* 2002, **99:**16587-16591.
